# Prognostic Impact of Tumor Size in Patients with Stage T3N1 Colon Cancer

**DOI:** 10.3390/jcm15010247

**Published:** 2025-12-29

**Authors:** Ezgi Turkoglu, Nisanur Sarıyar Busery, Sedat Yildirim, Goncagül Akdağ Topal, Cevher Burcu Salman, Erhan Conay, Furkan Turkoglu, Ozhan Albayrak, Seval Ay Ersoy, Deniz Isik, Hatice Odabaş, Cihad Tatar, Nedim Turan

**Affiliations:** 1 Department of Medical Oncology, Kartal Dr. Lütfi Kirdar City Hospital, Health Science University, 34865 İstanbul, Türkiye; 2 Department of Medical Oncology, Tokat State Hospital, 60100 Tokat, Türkiye; 3 Department of General Surgery, Aktif International Hospital, 41275 Kocaeli, Türkiye; 4 Department of General Surgery, Yozgat City Hospital, 66100 Yozgat, Türkiye; 5 Independent Researcher, Canberra, ACT 2605, Australia

**Keywords:** colon cancer, T3N1 stage, tumor size, prognosis, recurrence-free survival, adjuvant chemotherapy

## Abstract

**Background/Objectives**: Tumor size is not included in the TNM staging system for colon cancer, and its prognostic significance remains controversial. We aimed to evaluate the impact of tumor size on recurrence-free survival (RFS) and overall survival (OS) in patients with stage T3N1 colon cancer. **Methods**: We retrospectively analyzed 336 patients with pathologically confirmed pT3N1 colon cancer who underwent curative resection between January 2015 and January 2025 at our tertiary institution. Clinicopathological features, adjuvant chemotherapy details, and survival outcomes were collected. Tumor size was measured pathologically, and a cutoff was determined by receiver operating characteristic (ROC) analysis. Kaplan–Meier and Cox regression analyses were performed to identify prognostic factors. **Results**: The optimal cutoff for tumor size predicting recurrence was 4 cm. Patients with tumors ≥ 4 cm had significantly lower 5-year RFS compared to those with smaller tumors (65.1% vs. 80.3%, *p* = 0.007). In multivariate analysis, tumor size ≥ 4 cm (HR: 2.014, 95% CI: 1.093–3.714, *p* = 0.025), ECOG performance status ≥ 2 (*p* = 0.005), positive resection margin (*p* = 0.011), and failure to complete adjuvant chemotherapy (*p* = 0.007) were identified as independent adverse prognostic factors for RFS. Tumor size was not independently associated with OS (*p* = 0.46). Adjuvant chemotherapy significantly improved both RFS (*p* < 0.001) and OS (*p* < 0.001). **Conclusions**: In patients with stage T3N1 colon cancer, tumor size ≥ 4 cm is an independent adverse prognostic factor for RFS. Incorporating tumor size into risk stratification, alongside TNM staging and treatment completion status, may improve prognostic assessment and guide clinical decision-making.

## 1. Introduction

Colorectal cancer is a major global health issue and has been reported as the third most common malignancy in men and the second most common in women worldwide [1]. Locally advanced colorectal cancer accounts for approximately 5–22% of all colorectal cancer cases [2]. These tumors are classified using the Tumor–Node–Metastasis (TNM) staging system, which plays a key role in predicting disease prognosis and guiding appropriate treatment strategies [3].

In colorectal cancer, the T factor is determined by the depth of vertical invasion, while the prognostic importance of tumor size, which indicates horizontal extension, remains uncertain. Several studies in the literature have shown that larger tumor size may act as an unfavorable prognostic factor [4,5]. However, especially in stage IIA disease, smaller tumors have been found to behave more aggressively biologically and may be linked to worse survival outcomes than expected [6,7,8].

In addition, analyses of T4 tumors and node-positive (stage III) cases have shown that increasing tumor size independently negatively affects survival [9,10,11,12,13]. Therefore, there is a growing consensus that tumor size should be viewed not just as an anatomical measure, but also as an essential parameter that reflects tumor biology and the progression of the disease.

In colon cancer, adjuvant chemotherapy is recognized as the standard approach for improving survival, particularly in patients with stage III disease [14,15,16]. Large-scale studies conducted by the International Duration Evaluation of Adjuvant Therapy (IDEA) consortium defined T1-3N1 patients as “low-risk stage III”. However, although the T3N1 subgroup is categorized as low risk, evidence has shown that prognosis within this group is heterogeneous, with some patients experiencing poorer survival outcomes [17]. This heterogeneity highlights the limitation of relying solely on the TNM staging system to predict prognosis. In recent years, numerous studies have investigated the prognostic value of additional parameters, including tumor size, histopathological subtype, tumor grade, lymph node count, lymphovascular/perineural invasion, and microsatellite instability (MSI). Notably, although tumor size is incorporated into staging systems for many solid tumors, it has not been included in the TNM classification for colon cancer. Therefore, identifying additional prognostic factors in patients with stage T3N1 colon cancer is of considerable clinical importance for better predicting the risk of recurrence and for tailoring adjuvant treatment strategies.

Although the prognostic role of tumor size remains controversial in the literature, its significance within the T3N1 colon cancer subgroup has not been adequately elucidated. In this study, we aimed to evaluate the impact of tumor size on survival in patients with T3N1 colon cancer who underwent curative surgery.

## 2. Materials and Methods

### 2.1. Patients and Study Design

This retrospective study included patients who underwent surgical resection for colon cancer between January 2015 and January 2025, were pathologically staged as pT3N1, and subsequently presented to the Medical Oncology Department of Kartal Dr. Lutfi Kirdar City Hospital for treatment and follow-up. Patient demographic, clinicopathological, and oncological data were collected from patient files and electronic medical records.

**Figure 1:**
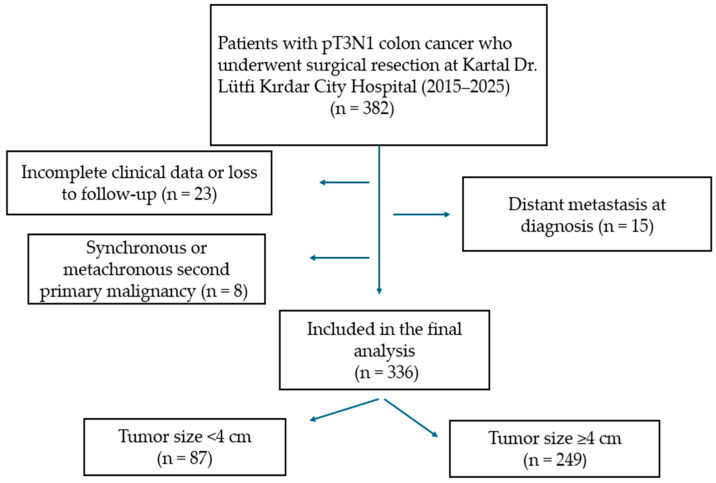
Flow diagram illustrating patient selection, exclusion criteria, and stratification according to tumor size.

Patients who were histologically diagnosed with T3N1 colon cancer according to the 8th edition of the TNM staging system and underwent curative surgery were included in the study. Patients were excluded if they had distant metastasis at diagnosis, received neoadjuvant chemotherapy or radiotherapy, were diagnosed with rectal cancer, had a secondary malignancy, or had incomplete clinical or pathological data. A flow diagram illustrating the patient selection and exclusion process is provided in Figure 1.

The study protocol was approved by the Ethics Committee of Kartal Dr. Lutfi Kirdar City Hospital (Approval No: 2025/010.99/1715, Date: 25 June 2025). As the study was based on a retrospective review of medical records, the requirement for informed consent was waived by the ethics committee.

### 2.2. Adjuvant Chemotherapy and Follow-Up

Adjuvant chemotherapy was administered as combination regimens in patients with adequate performance status and no medical contraindications to oxaliplatin. In cases with oxaliplatin contraindications or relatively poor performance status, single-agent therapy was preferred. The treatment protocols included modified FOLFOX (folinic acid, 5-fluorouracil, and oxaliplatin), XELOX (capecitabine and oxaliplatin), and capecitabine monotherapy. Adjuvant chemotherapy regimens were planned in accordance with the contemporary clinical guidelines relevant to the treatment period, taking into account physician discretion and patient tolerance.

As part of the follow-up protocol, patients underwent a physical examination and a review of their medical history every 3–6 months during the first 2 years, and every 6 months thereafter for a total of 5 years. In addition, thoracic, abdominal, and pelvic CT imaging was performed every 6–12 months for 5 years postoperatively.

### 2.3. Data Collection and Outcome Measures

Collected variables included age, sex, tumor location (right/left colon), histological subtype, histological grade, TNM classification, number of lymph nodes retrieved, status of lymphovascular and perineural invasion, microsatellite instability (MSI) status, date of surgery, and tumor size. Oncological data recorded were receipt of adjuvant chemotherapy, regimen type, treatment duration, treatment completion status, disease recurrence, and date of death.

All pathological evaluations, including tumor size measurement, histological subtype classification, grading, and MSI assessment, were performed at our institution. Tumor diameter was defined as the maximum dimension of the fixed surgical specimen measured macroscopically at pathological examination and was assessed to include both invasive and non-invasive components. Histological classification was conducted according to the World Health Organization (WHO) classification. MSI status was evaluated using immunohistochemical analysis.

Staging was performed according to the 8th edition of the UICC TNM classification [18]. Tumor diameter was defined as the maximum dimension of the fixed specimen at pathological examination and encompassed the entire lesion, including both invasive and non-invasive components. The colon segments from the cecum to the hepatic flexure and the proximal half of the transverse colon were classified as the right colon. In contrast, the segments from the splenic flexure to the rectosigmoid region and the distal half of the transverse colon were classified as the left colon. Overall survival (OS) was defined as the time from the date of surgery to death from any cause, while recurrence-free survival (RFS) was defined as the time from the date of surgery to the first documented recurrence (local, regional, or distant metastasis) or death from any cause, whichever occurred first. Patients without recurrence who were alive were considered alive and recurrence-free at the date of last follow-up. The primary endpoint of the study was RFS, and the secondary endpoint was OS.

### 2.4. Statistical Analysis

Statistical analyses were performed using SPSS software (IBM Corp., Armonk, NY, USA; version 27). Descriptive statistics were presented as mean ± standard deviation or median (minimum–maximum) for continuous variables, and as frequencies and percentages for categorical variables. The distribution of continuous variables was assessed using the Shapiro–Wilk test. Comparisons between groups were conducted using the Chi-square or Fisher’s exact test for categorical variables, and Student’s *t*-test for continuous variables when parametric assumptions were met, or the Mann–Whitney U test when they were not.

The optimal cutoff value for tumor size was determined using receiver operating characteristic (ROC) curve analysis. Survival analyses were performed using the Kaplan–Meier method, and differences between groups were assessed with the log-rank test. To identify prognostic factors, univariate and multivariate Cox regression analyses were conducted, and the results were reported as hazard ratios (HRs) with 95% confidence intervals (CIs). A *p*-value of <0.05 was considered statistically significant.

## 3. Results

### 3.1. Baseline Clinicopathological Characteristics

A total of 336 patients were included in the study. The median age was 65 years (range: 25–86), with 51.5% younger than 65 years and 48.5% aged 65 years or older. Regarding sex distribution, 60.1% of the patients were male and 39.9% were female. In terms of performance status, 88.7% of patients had an ECOG-PS (Eastern Cooperative Oncology Group-Performance Status) of 0–1, while 11.3% had an ECOG-PS of 2.

Regarding tumor location, 30.7% of cases were in the right colon, while 69.3% were in the left colon. Lymphovascular invasion was identified in 58.6% of patients and perineural invasion in 42.9%. According to histological grade, 11.9% of tumors were classified as grade 1, 83.9% as grade 2, and 4.2% as grade 3. In terms of histological subtype, 88.1% of cases were adenocarcinomas and 11.9% were mucinous adenocarcinomas.

According to MSI status, 76.2% of patients were pMMR (proficient mismatch repair), 5.7% were dMMR (deficient mismatch repair), while MSI status was unknown in 18.2%. Evaluation of the number of lymph nodes retrieved showed that fewer than 12 lymph nodes were removed in 9.5% of patients, whereas 12 or more lymph nodes were removed in 90.5% of patients.

In terms of surgical resection, R0 resection was achieved in 97.9% of cases, while 2.1% underwent R1 resection. Adjuvant chemotherapy was administered to 93.2% of patients, whereas 6.8% did not receive it. Among the 313 patients who received adjuvant chemotherapy, 72.8% were treated with XELOX (capecitabine + oxaliplatin), 13.4% with FOLFOX (folinic acid + 5-FU + oxaliplatin), and 13.7% with capecitabine monotherapy. Of these patients, 91.1% completed the planned treatment, while 8.9% did not.

In the study population, the median tumor size was 4.9 cm, ranging from 0.9 to 12.0 cm. The median follow-up duration was 53 months (95% CI: 48.6–57.4). The general clinicopathological characteristics of the patients are summarized in Table 1.

### 3.2. Comparison of Clinicopathological Characteristics According to Tumor Size

To evaluate the predictive value of tumor size for postoperative recurrence, the optimal cutoff was determined through ROC curve analysis, and a threshold of 4 cm was identified (AUC = 0.572, 95% CI: 0.505–0.640; *p* = 0.042).

With respect to histological subtype, mucinous adenocarcinoma was observed more frequently in patients with tumors ≥ 4 cm compared with those with tumors < 4 cm (14.9% vs. 3.4%; *p* = 0.003). Regarding histological grade, grade 3 tumors were identified exclusively in the ≥4 cm tumor group (5.6% vs. 0%; *p* = 0.049).

In terms of tumor location, right-sided colon tumors were significantly more common in the ≥4 cm group than in the <4 cm group (34.1% vs. 20.7%; *p* = 0.022).

No significant differences were observed between the two groups with respect to lymphovascular invasion (*p* = 0.80), perineural invasion (*p* = 0.50), MSI status (*p* = 0.97), number of retrieved lymph nodes (*p* = 0.25), resection margin status (*p* = 1.00), or N substage distribution (*p* = 0.179).

When demographic and treatment-related variables were evaluated, no significant differences were observed between the groups with respect to sex (*p* = 0.70) or ECOG performance status (*p* = 0.33). The proportion of patients younger than 65 years was higher in the <4 cm tumor group (60.9% vs. 48.2%; *p* = 0.046). In addition, adjuvant chemotherapy use differed between the groups, with a higher proportion of patients receiving adjuvant treatment in the <4 cm tumor group (98.9% vs. 91.2%; *p* = 0.015).

### 3.3. Prognostic Impact of Tumor Diameter on Recurrence-Free and Overall Survival

**Figure 2:**
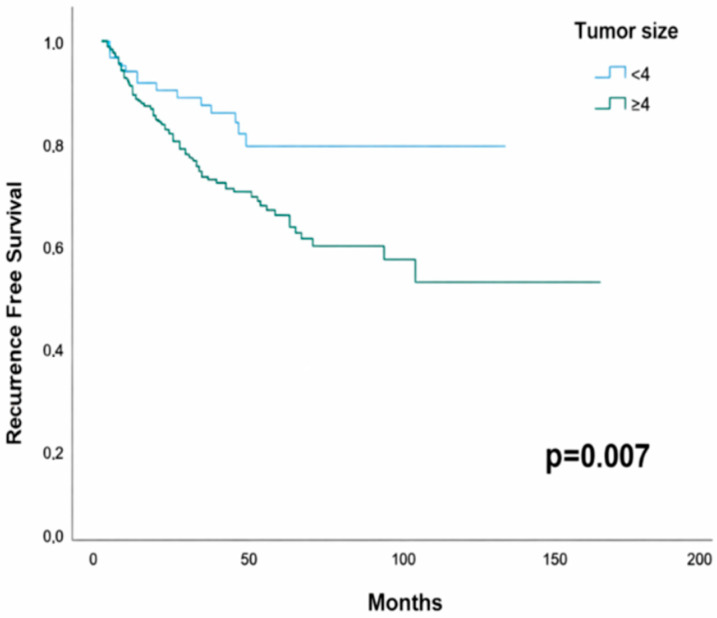
Kaplan–Meier curves for recurrence-free survival according to tumor size (<4 cm vs. ≥4 cm).

In the entire cohort, the 5-year RFS rate was 70.2% (95% CI: 64.7–75.7). The 5-year RFS was 80.3% (95% CI: 70.7–89.9) in patients with tumors < 4 cm, compared to 65.1% (95% CI: 58.0–72.1) in those with tumors ≥ 4 cm; this difference was statistically significant (*p* = 0.007; Figure 2).

In Cox regression analysis, a tumor size of ≥4 cm was associated with an increased risk of recurrence (HR:2.15, 95% CI:1.21–3.80; *p* = 0.009). No significant difference in overall survival (OS) was observed between tumor size groups.

**Figure 3:**
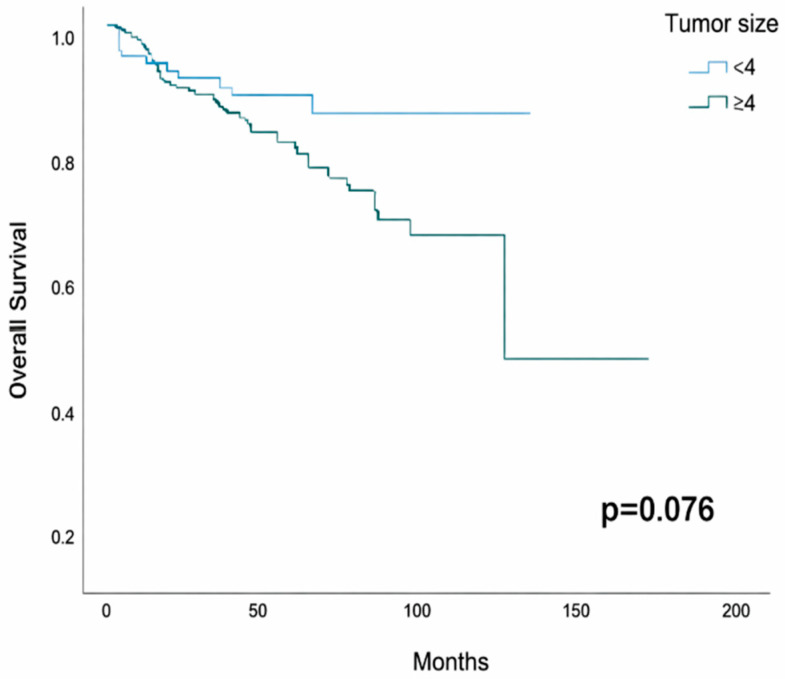
Kaplan–Meier curves for overall survival according to tumor size (<4 cm vs. ≥4 cm).

The 5-year OS was 84.6% (95% CI: 75.8–93.4) in patients with tumors < 4 cm and 78.8% (95% CI: 72.7–84.9) in those with tumors ≥ 4 cm (*p* = 0.076; Figure 3).

### 3.4. Association Between Clinicopathological Factors and Recurrence-Free Survival

In univariate Cox regression analysis, ECOG performance status ≥ 2 (HR: 2.546; 95% CI: 1.516–4.278; *p* < 0.001), positive resection margin (HR: 2.783; 95% CI: 1.018–7.607; *p* = 0.046), tumor size ≥ 4 cm (HR: 2.148; 95% CI: 1.213–3.801; *p* = 0.009), and failure to complete adjuvant chemotherapy (HR: 1.422; 95% CI: 1.019–1.984; *p* = 0.038) were significantly associated with shorter RFS. Conversely, receipt of adjuvant chemotherapy was strongly associated with longer recurrence-free survival (HR: 0.270; 95% CI: 0.157–0.465; *p* < 0.001). In univariate Cox regression analysis, neither N1b (HR: 0.89; 95% CI: 0.45–1.77; *p* = 0.747) nor N1c (HR: 0.86; 95% CI: 0.43–1.72; *p* = 0.672) showed a significant difference in recurrence-free survival compared with N1a. Likewise, compared with patients without positive lymph nodes, having 1 (HR: 0.93; 95% CI: 0.41–2.08; *p* = 0.852), 2 (HR: 0.75; 95% CI: 0.45–1.23; *p* = 0.253), or 3 positive lymph nodes (HR: 0.63; 95% CI: 0.28–1.01; *p* = 0.062) was not significantly associated with recurrence-free survival.

In multivariate Cox regression analysis, ECOG performance status ≥ 2 (HR: 2.444; 95% CI: 1.301–4.591; *p* = 0.005), positive resection margin (HR: 3.769; 95% CI: 1.361–10.436; *p* = 0.011), tumor size ≥ 4 cm (HR: 2.014; 95% CI: 1.093–3.714; *p* = 0.025), and failure to complete adjuvant chemotherapy (HR: 1.595; 95% CI: 1.134–2.243; *p* = 0.007) were identified as independent adverse prognostic factors. Administration of adjuvant chemotherapy, on the other hand, was determined to be an independent protective factor for RFS (HR: 0.119; 95% CI: 0.035–0.408; *p* < 0.001) (Table 2).

### 3.5. Association Between Clinicopathological Factors and Overall Survival

In univariate Cox regression analysis, age ≥ 65 years (HR: 1.838; 95% CI: 1.113–3.034; *p* = 0.017), ECOG performance status ≥ 2 (HR: 3.710; 95% CI: 2.080–6.616; *p* < 0.001), positive resection margin (HR: 3.884; 95% CI: 1.409–10.705; *p* = 0.009), and failure to complete adjuvant chemotherapy (HR: 2.079; 95% CI: 1.482–2.915; *p* < 0.001) were identified as prognostic factors adversely affecting overall survival. In contrast, receipt of adjuvant chemotherapy (HR: 0.192; 95% CI: 0.107–0.345; *p* < 0.001) was found to be a significant protective factor that improved overall survival (Table 3). In univariate Cox regression analysis, compared with N1a, neither N1b (HR: 1.37; 95% CI: 0.54–3.50; *p* = 0.508) nor N1c (HR: 1.09; 95% CI: 0.42–2.87; *p* = 0.855) was significantly associated with overall survival. Likewise, compared with patients without positive lymph nodes, having 1 (HR: 1.12; 95% CI: 0.40–3.19; *p* = 0.827), 2 (HR: 1.01; 95% CI: 0.52–1.94; *p* = 0.987), or 3 positive lymph nodes (HR: 0.74; 95% CI: 0.33–1.65; *p* = 0.459) was not significantly associated with overall survival.

In multivariate Cox regression analysis, ECOG-PS ≥ 2 (HR: 3.656; 95% CI: 1.687–7.922; *p* = 0.001), positive resection margin (HR: 5.834; 95% CI: 2.056–16.555; *p* < 0.001), and failure to complete adjuvant chemotherapy (HR: 2.398; 95% CI: 1.682–3.420; *p* < 0.001) were identified as independent adverse prognostic factors. Conversely, receipt of adjuvant chemotherapy (HR: 0.095; 95% CI: 0.033–0.274; *p* < 0.001) emerged as an independent protective factor significantly associated with improved overall survival (Table 3).

## 4. Discussion

Risk assessment in colorectal cancer is generally based on the TNM staging system, which describes disease extent according to the depth of primary tumor invasion (T), lymph node involvement (N), and the presence of distant metastasis (M). However, while the T category reflects the vertical depth of tumor invasion through the bowel wall, evidence regarding the biological significance of horizontal extension, as measured by tumor diameter, remains limited and conflicting. This uncertainty underscores the need for further investigation into the prognostic value of tumor size and its role in clinical decision-making. In this study, we evaluated the prognostic significance of tumor size in patients with stage T3N1 colon cancer who underwent surgery.

There is currently no universally accepted cutoff value for tumor size in international guidelines, and the thresholds used in prior studies remain highly heterogeneous. Several investigations, particularly in stage II–III disease, have reported that a <4 cm/≥4 cm tumor size distinction may hold prognostic relevance [19,20,21]. In line with this heterogeneity, we performed a ROC curve analysis within our cohort to identify the optimal discriminatory threshold, and a cutoff value of 4 cm was determined to be the most appropriate point. Therefore, the 4 cm threshold used in our study is supported both by existing literature and by our cohort-specific statistical analysis.

Several studies, consistent with our findings, have demonstrated that larger tumor size is associated with poorer prognosis. For instance, in patients with tumors ≥ 5 cm, 5-year OS (63.5% vs. 75.2%) and RFS (59.5% vs. 72.4%) were significantly lower, and in multivariate analysis, tumor size was confirmed as an independent adverse prognostic factor for both OS (HR: 1.43; *p* = 0.014) and RFS (HR: 1.45; *p* = 0.007) [10]. Kornprat et al. reported lower disease-free survival (DFS) (50% vs. 65%) and cancer-specific survival (CSS) (55% vs. 72%) in patients with tumors > 4.5 cm, and multivariate analyses supported the independent prognostic value of tumor size [5]. These findings suggest that increasing tumor size may reflect a greater tumor burden, a higher risk of micrometastatic disease, and more aggressive biological behavior. Indeed, a meta-analysis has also reported that tumor size ≥ 4 cm, particularly when accompanied by other adverse pathological features or incomplete surgical margins, may be associated with poor prognosis [19]. A recent large-scale SEER analysis of patients with small bowel adenocarcinoma similarly demonstrated that tumor size, particularly ≥3–4 cm, was an independent adverse prognostic factor for survival [22]. These findings suggest that tumor diameter may exert a similar prognostic effect across different gastrointestinal tumors.

However, evidence in the literature also suggests that tumor size may demonstrate an inverse association with prognosis, particularly in low-risk groups. In stage IIA (T3N0) patients, a tumor diameter of <4 cm was found to be associated with lower 5-year DFS compared with tumors ≥ 4 cm (71.7% vs. 87.6%; *p* = 0.028) [8]. Nevertheless, the absence of adjuvant chemotherapy in that study suggests that the unfavorable outcomes observed in smaller tumors may be attributable to biological aggressiveness rather than lack of treatment. In our T3N1 cohort, by contrast, larger tumor size was associated with poorer prognosis. This discrepancy likely reflects differences in biological behavior between node-negative and node-positive patients, as well as the defining impact of tumor burden in stage III disease. Consistently, in another T3N0 cohort, tumors < 5 cm were identified as an independent adverse prognostic factor (HR: 3.11; 95% CI: 1.33–7.30; *p* = 0.009) [8]. The absence of MSI data in that study represents a significant limitation, as it is well established that MSI-H tumors are associated with a more favorable prognosis, whereas MSS tumors exhibit poorer outcomes [23]. Therefore, the poor prognosis observed in smaller tumors may have resulted from differences in MSI distribution rather than true biological aggressiveness. In another SEER-based analysis, tumors ≤ 2.5 cm were found to be associated with lower 8-year CSS, whereas larger tumors were reported to carry a lower risk of mortality (HR: 0.74–0.77; *p* < 0.02) [7]. However, this study did not include RFS data or information on adjuvant chemotherapy, and the results may therefore have been influenced by differences in treatment and heterogeneity in follow-up.

Muralidhar et al. [9] reported that in node-positive colon cancer, very small tumors (<5 mm) were associated with a higher risk of mortality, intermediate-sized tumors (5–59 mm) had more favorable outcomes, and tumors ≥ 60 mm were again associated with increased risk. This finding suggests that the relationship between tumor size and survival in node-positive cases may not be linear, with both biologically aggressive, very small tumors and large tumors exerting adverse prognostic effects.

In a study conducted in stage T4 disease, a tumor size of <50 mm was identified as an independent adverse prognostic factor for both DFS (*p* = 0.009) and CSS (*p* = 0.011) [11]. In our T3N1 cohort, by contrast, a tumor size of ≥4 cm was identified as an adverse prognostic factor for RFS. The association of smaller tumor size (<50 mm) with poor prognosis in the T4 study may be attributable to the inclusion of a heterogeneous patient population (N0–N2) and the possibility that small lesions in T4 tumors represent a biologically more aggressive phenotype. This discrepancy suggests that the prognostic impact of tumor size may vary according to both T stage and nodal status.

In another study of patients with T4bN0–2M0 disease, smaller tumors were unexpectedly associated with worse CSS (*p* < 0.001). In this analysis, the risk of mortality was 18% lower in tumors measuring 4–7 cm and 22% lower in those ≥ 7 cm [12]. However, the study evaluated only CSS, with no reporting of DFS or RFS, and did not provide information on adjuvant chemotherapy, MSI, or BRAF mutation status. Moreover, significance in nodal subgroup analyses was demonstrated only in T4bN0 patients. The strength of our study lies in its entirely homogeneous T3N1 cohort, which allows for meaningful comparisons across tumor size subgroups.

Tamai et al. reported that in 162 patients with pT3N1 colon cancer, a tumor size of <45 mm was significantly associated with poorer OS, RFS, and CSS, and was identified as an independent prognostic factor [13]. However, this study did not report adjuvant chemotherapy regimens or treatment completion rates, nor did it provide information on MSI or BRAF mutation status. These methodological limitations may have contributed to the differences in outcomes compared with our study. In contrast, the relatively larger sample size in our cohort enhances the reliability of our findings and strengthens their contribution to the literature.

Our study also evaluated the impact of resection status on prognosis. In univariate analyses, R1 resection was significantly associated with poorer outcomes in terms of both RFS and OS. This effect persisted in multivariate Cox regression analysis, indicating that R1 resection is an independent adverse prognostic factor, irrespective of other clinicopathological variables. This finding is consistent with results reported in the literature [2,24].

The efficacy of adjuvant chemotherapy in stage III colon cancer was first demonstrated by Moertel et al., showing that the combination of 5-FU and levamisole was superior to surgery alone with observation. This finding established the foundation of adjuvant therapy [25]. Subsequent large-scale studies have confirmed the superiority of 5-FU/LV (5- fluorouracil/leucovorin) over surgery alone [16,26]. In the modern era, the MOSAIC and NSABP C-07 trials demonstrated that the addition of oxaliplatin to existing 5-FU/LV-based standards further improved survival [14,27]. Ultimately, the IDEA consortium demonstrated that three months of XELOX is sufficient for patients with low-risk stage III disease, whereas six months of treatment is more appropriate for those in the high-risk group [17]. In our study, only patients with T3N1 (low-risk) disease were included, and the administration of adjuvant chemotherapy was identified as a strong and independent protective factor for RFS (HR: 0.119; 95% CI: 0.035–0.408; *p* < 0.001). This finding fills an important gap in the literature with real-world data, particularly as it includes patients who did not receive chemotherapy as well as those who did not complete treatment. However, since patient enrollment began in 2015, the IDEA results had not yet been published at that time, and treatment duration was determined according to physician preference.

The inability of tumor size to predict OS despite its significant association with RFS may reflect the natural course of T3N1 colon cancer, in which recurrence events occur earlier and thus more strongly influence RFS. Effective post-recurrence therapies may attenuate survival differences over time. Moreover, the median follow-up of 53 months may have been insufficient to capture all OS events, potentially limiting the ability to detect an association with overall survival.

Our study has several limitations. First, the retrospective and single-center design may introduce selection bias and limit the homogeneity of the data. The relatively limited median follow-up duration may also have hindered the complete assessment of long-term survival outcomes. Tumor size was defined as the maximum diameter of the fixed resected specimen based on macroscopic measurements reported in pathology records, encompassing both invasive and non-invasive components. As the invasive tumor diameter was not consistently documented for all cases, analyses restricted to the invasive component could not be performed. Although dichotomization of tumor size (<4 cm vs. ≥4 cm) is clinically practical, it may have obscured potential non-linear associations between tumor size and prognosis; non-linear Cox regression models were therefore not applied due to the limited number of events. In addition, the ROC analysis used to determine the tumor size cut-off was based on recurrence assessed as a binary outcome, which entails methodological limitations for time-dependent survival endpoints, and the ROC results should be interpreted as supportive rather than definitive. Moreover, important molecular markers, such as KRAS, NRAS, and BRAF mutations, could not be evaluated, thereby restricting the ability to analyze the impact of tumor biology on survival comprehensively. In addition, several potential prognostic factors, including emergency presentation (obstruction or perforation), baseline carcinoembryonic antigen levels, comorbidities, detailed T3 invasion depth, extramural vascular invasion, and tumor budding, were not available in a standardized manner due to the retrospective nature of the study and therefore could not be included in the analyses. Finally, heterogeneity among adjuvant treatment regimens made it difficult to assess treatment response uniformly. Nevertheless, despite these limitations, the findings are considered complementary to the existing literature and of potential value to clinical practice.

## 5. Conclusions

In conclusion, in our cohort of patients with stage T3N1 colon cancer, a tumor size of ≥4 cm was identified as an independent adverse prognostic factor for RFS. While smaller tumor size has been associated with poorer outcomes in node-negative stage II subgroups, the more substantial impact of larger tumors in node-positive stage III disease suggests that the prognostic value of tumor size may vary according to stage and nodal status. Our results indicate that incorporating tumor diameter into prognostic assessment alongside stage and nodal status may improve the accuracy of clinical risk stratification. Furthermore, the strength of our study lies in its homogeneous and relatively large cohort of exclusively T3N1 patients, the inclusion of real-world data on adjuvant chemotherapy, and its ability to address the methodological shortcomings of prior studies, thereby providing a meaningful contribution to the literature.

## Figures and Tables

**Table 1:** Clinicopathological characteristics of patients according to tumor size.

Variable	Subgroups	Total	Small Tumor <4 cm (*n* = 87)	Large Tumor ≥4 cm (*n* = 249)	*p*
Age	<65 years	173 (51.5%)	53 (60.9%)	120 (48.2%)	***0.046***
	≥65 years	163 (48.5%)	34 (39.1%)	129 (51.8%)	
Sex	Female	134 (39.9%)	33 (37.9%)	101 (40.6%)	0.70
	Male	202 (60.1%)	54 (62.1%)	148 (59.4%)	
ECOG-PS	0–1	298 (88.7%)	80 (92.0%)	218 (87.6%)	0.33
	2	38 (11.3%)	7 (8.0%)	31 (12.4%)	
Tumor Location	Right colon	103 (30.7%)	18 (20.7%)	85 (34.1%)	***0.022***
	Left colon	233 (69.3%)	69 (79.3%)	164 (65.9%)	
LVI	Present	197 (58.6%)	50 (57.5%)	147 (59.0%)	0.80
	Absent	139 (41.4%)	37 (42.5%)	102 (41.0%)	
PNI	Present	144 (42.9%)	40 (46.0%)	104 (41.8%)	0.50
	Absent	192 (57.1%)	47 (54.0%)	145 (58.2%)	
Tumor Grade	Grade 1	40 (11.9%)	13 (14.9%)	27 (10.8%)	***0.049***
	Grade 2	282 (83.9%)	74 (85.1%)	208 (83.5%)	
	Grade 3	14 (4.2%)	0 (0%)	14 (5.6%)	
Histological Subtype	Adenocarcinoma, NOS	296 (88.1%)	84 (96.6%)	212 (85.1%)	***0.003***
	Mucinous adenocarcinoma	40 (11.9%)	3 (3.4%)	37 (14.9%)	
MSI Status	pMMR	256 (76.2%)	67 (77.0%)	189 (75.9%)	0.97
	dMMR	19 (5.7%)	5 (5.7%)	14 (5.6%)	
	Unknown	61 (18.2%)	15 (17.2%)	46 (18.5%)	
N1 substage	N1a	158 (47.0%)	47 (29.7%)	111 (70.3%)	0.179
	N1b	145 (43.2%)	35 (24.1%)	110 (75.9%)	
	N1c	33 (9.8%)	5 (15.2%)	28 (84.8%)	
Number of Retrieved Lymph Nodes	<12	32 (9.5%)	11 (12.6%)	21 (8.4%)	0.25
	≥12	304 (90.5%)	76 (87.4%)	228 (91.6%)	
Resection Status	R0	329 (97.9%)	85 (97.7%)	244 (98.0%)	1.000
	R1	7 (2.1%)	2 (2.3%)	5 (2.0%)	
Adjuvant Chemotherapy	Received	313 (93.2%)	86 (98.9%)	227 (91.2%)	***0.015***
	Not received	25 (6.8%)	1 (1.1%)	22 (8.8%)	
Adjuvant Chemotherapy Regimen (*n* = 313)	FOLFOX	42 (13.4%)	5 (5.8%)	37 (16.3%)	0.05
	XELOX	228 (72.8%)	69 (80.2%)	159 (70.0%)	
	Capecitabine	43 (13.7%)	12 (14.0%)	31 (13.7%)	
Completion of Adjuvant Chemotherapy (*n* = 313)	Completed	285 (91.1%)	78 (91.8%)	207 (90.8%)	0.79
	Not completed	28 (8.9%)	7 (8.2%)	21 (9.2%)	

ECOG-PS—Eastern Cooperative Oncology Group performance status; LVI—lymphovascular invasion; PNI—perineural invasion; MSI—microsatellite instability; dMMR—deficient mismatch repair; pMMR—proficient mismatch repair; R0—microscopically margin-negative resection; R1—microscopically margin-positive resection; FOLFOX—5-fluorouracil, leucovorin, and oxaliplatin; XELOX—capecitabine and oxaliplatin. NOS: not otherwise specified. Bold and italics indicate significant *p* values.

**Table 2:** Univariate and multivariate analysis in recurrence-free survival.

	Univariate Analysis			Multivariate Analysis		
Characteristics	HR	95% Cl	*p*	HR	95% Cl	*p*
Sex (Female vs. Male)	1.341	0.871–2.066	0.183			
Age (<65 vs. ≥65)	1.369	0.904–2.073	0.138			
ECOG-PS (0–1 vs. ≥2)	2.546	1.516–4.278	***<0.001***	2.444	1.301–4.591	***0.005***
Tumor grade (Grade 1 + 2 vs. Grade 3)	1.048	0.634–1.733	0.854			
Histological subtype (Adenocarcinoma, NOS vs. Mucinous adenocarcinoma)	1.220	0.689–2.159	0.495			
LVI (Present vs. Absent)	1.264	0.828–1.929	0.274			
PNI (Present vs. Absent)	1.200	0.791–1.820	0.392			
MSI status (dMMR vs. pMMR)	1.023	0.804–1.303	0.851			
Tumor location (Right vs. Left colon)	1.141	0.738–1.763	0.553			
Resection status (R0 vs. R1)	2.783	1.018–7.607	***0.046***	3.769	1.361–10.436	***0.011***
Tumor size (<4 cm vs. ≥4 cm)	2.148	1.213–3.801	***0.009***	2.014	1.093–3.714	***0.025***
Number of retrieved lymph nodes (<12 vs. ≥12)	1.425	0.688–2.952	0.341			
Adjuvant chemotherapy (Yes vs. No)	0.270	0.157–0.465	***<0.001***	0.119	0.035–0.408	***<0.001***
Adjuvant chemotherapy regimen (FOLFOX XELOX vs. Capecitabine)	1.207	0.934–1.560	0.151			
Completion of adjuvant chemotherapy (Completed vs. not completed)	1.422	1.019–1.984	***0.038***	1.595	1.134–2.243	***0.007***

LVI—lymphovascular invasion; PNI—perineural invasion; HR—hazard ratio; CI—confidence interval; ECOG-PS—Eastern Cooperative Oncology Group performance status; MSI—microsatellite instability; dMMR—deficient mismatch repair; pMMR—proficient mismatch repair. R0—microscopically margin-negative resection; R1—microscopically margin-positive resection; FOLFOX—5-fluorouracil, leucovorin, and oxaliplatin; XELOX—capecitabine and oxaliplatin; NOS—not otherwise specified. Bold and italics indicate significant *p* values.

**Table 3:** Univariate and multivariate analysis in overall survival.

	Univariate Analysis			Multivariate Analysis		
Characteristics	HR	95% Cl	*p*	HR	95% Cl	*p*
Sex (Female vs. Male)	1.207	0.728–2.004	0.47			
Age (<65 vs. ≥65)	1.838	1.113–3.034	***0.017***	1.061	0.563–1.998	0.86
ECOG-PS (0–1 vs. ≥2)	3.710	2.080–6.616	***<0.001***	3.656	1.687–7.922	***0.001***
Tumor grade (Grade 1 + 2 vs. Grade 3)	0.993	0.554–1.781	0.98			
Histological subtype (Adenocarcinoma, NOS vs. Mucinous adenocarcinoma)	0.843	0.401–1.773	0.65			
LVI (Present vs. Absent)	0.871	0.533–1.425	0.58			
PNI (Present vs. Absent)	0.851	0.513–1.413	0.53			
MSI status (dMMR vs. pMMR)	1.228	0.993–1.617	0.14			
Tumor location (Right vs. Left colon)	1.562	0.948–2.573	0.08			
Resection status (R0 vs. R1)	3.884	1.409–10.705	***0.009***	5.834	2.056–16.555	***<0.001***
Tumor size (<4 cm vs. ≥4 cm)	1.784	0.931–3.417	0.08	1.296	0.653–2.572	0.46
Number of retrieved lymph nodes (<12 vs. ≥12)	1.462	0.628–3.405	0.38			
Adjuvant chemotherapy (Yes vs. No)	0.192	0.107–0.345	***<0.001***	0.095	0.033–0.274	***<0.001***
Adjuvant chemotherapy regimen (FOLFOX XELOX vs. Capecitabine)	1.155	0.847–1.576	0.363			
Completion of adjuvant chemotherapy (Completed vs. not completed	2.079	1.482–2.915	***<0.001***	2.398	1.682–3.420	***<0.001***

LVI—lymphovascular invasion; PNI—perineural invasion; HR—hazard ratio; CI—confidence interval; ECOG-PS—Eastern Cooperative Oncology Group performance status; MSI—microsatellite instability; dMMR—deficient mismatch repair; pMMR—proficient mismatch repair; R0—microscopically margin-negative resection; R1—microscopically margin-positive resection; FOLFOX—5-fluorouracil, leucovorin, and oxaliplatin; XELOX—capecitabine and oxaliplatin. NOS—not otherwise specified. Bold and italics indicate significant *p* values.

## Data Availability

The data that support the findings of this study are available on request from the corresponding author.

## Abbreviations

CI	Confidence interval
CSS	Cancer-specific survival
DFS	Disease-free survival
dMMR	Deficient mismatch repair
ECOG-PS	Eastern Cooperative Oncology Group performance status
FOLFOX	5-fluorouracil, leucovorin, and oxaliplatin
HR	Hazard ratio
IDEA	International Duration Evaluation of Adjuvant Therapy
LN	Lymph node
LVI	Lymphovascular invasion
MSI	Microsatellite instability
MSI-H	High-frequency microsatellite instability
MSS	Microsatellite stable
OS	Overall survival
pMMR	Proficient mismatch repair
PNI	Perineural invasion
R0	Microscopically margin-negative resection
R1	Microscopically margin-positive resection
RFS	Recurrence-free survival
ROC	Receiver operating characteristic
XELOX	Capecitabine and oxaliplatin

## References

[1] International Agency for Research on Cancer, World Health Organization Global Cancer Observatory: Lyon, France, 2025. http://gco.iarc.fr/en.

[2] Campos F.G., Calijuri-Hamra M.C., Imperiale A.R., Kiss D.R., Nahas S.C., Cecconello I. (2011). Locally advanced colorectal cancer: Results of surgical treatment and prognostic factors. Arq. Gastroenterol..

[3] Amin M.B., Edge S.B., Greene F.L., Byrd D.R., Brookland R.K., Washington M.K., Gershenwald J.E., Compton C.C., Hess K.R., Sullivan D.C. (2017). AJCC Cancer Staging Manual.

[4] Saha S., Shaik M., Johnston G., Saha S.K., Berbiglia L., Hicks M., Gernand J., Grewal S., Arora M., Wiese D. (2015). Tumor size predicts long-term survival in colon cancer: An analysis of the National Cancer Data Base. Am. J. Surg..

[5] Kornprat P., Pollheimer M.J., Lindtner R.A., Schlemmer A., Rehak P., Langner C. (2011). Value of tumor size as a prognostic variable in colorectal cancer: A critical reappraisal. Am. J. Clin. Oncol..

[6] Lee S.Y., Kim C.H., Kim Y.J., Kim H.R. (2018). Macroscopic serosal invasion and small tumor size as independent prognostic factors in stage IIA colon cancer. Int. J. Color. Dis..

[7] Wang Y., Zhuo C., Shi D., Zheng H., Xu Y., Gu W., Cai S., Cai G. (2015). Unfavorable effect of small tumor size on cause-specific survival in stage IIA colon cancer, a SEER-based study. Int. J. Color. Dis..

[8] Santullo F., Biondi A., Cananzi F.C.M., Fico V., Tirelli F., Ricci R., Rizzo G., Coco C., Mattana C., D’Ugo D. (2018). Tumor size as a prognostic factor in patients with stage IIa colon cancer. Am. J. Surg..

[9] Muralidhar V., Nipp R.D., Ryan D.P., Hong T.S., Nguyen P.L., Wo J.Y. (2016). Association Between Very Small Tumor Size and Increased Cancer-Specific Mortality in Node-Positive Colon Cancer. Dis. Colon Rectum.

[10] Liang Y., Li Q., He D., Chen Y., Li J. (2021). Tumor size improves the accuracy of the prognostic prediction of T4a stage colon cancer. Sci. Rep..

[11] Shiraishi T., Ogawa H., Katayama A., Osone K., Okada T., Enokida Y., Oyama T., Sohda M., Shirabe K., Saeki H. (2022). Association of tumor size in pathological T4 colorectal cancer with desmoplastic reaction and prognosis. Ann. Gastroenterol. Surg..

[12] Huang B., Feng Y., Mo S.-B., Cai S.-J., Huang L.-Y. (2016). Smaller tumor size is associated with poor survival in T4b colon cancer. World J. Gastroenterol..

[13] Tamai K., Tei M., Tsujimura N., Nishida K., Mori S., Yoshikawa Y., Nomura M., Hamakawa T., Takiuchi D., Tsujie M. (2025). Impact of Small Tumor Size on Prognosis in T3N1 Colon Cancer. World J. Surg..

[14] André T., Boni C., Mounedji-Boudiaf L., Navarro M., Tabernero J., Hickish T., Topham C., Zaninelli M., Clingan P., Bridgewater J. (2004). Oxaliplatin, fluorouracil, and leucovorin as adjuvant treatment for colon cancer. N. Engl. J. Med..

[15] Twelves C., Wong A., Nowacki M.P., Abt M., Burris H.I., Carrato A., Cassidy J., Cervantes A., Fagerberg J., Georgoulias V. (2005). Capecitabine as adjuvant treatment for stage III colon cancer. N. Engl. J. Med..

[16] Haller D.G., Tabernero J., Maroun J., de Braud F., Price T., Van Cutsem E., Hill M., Gilberg F., Rittweger K., Schmoll H.-J. (2011). Capecitabine plus oxaliplatin compared with fluorouracil and folinic acid as adjuvant therapy for stage III colon cancer. J. Clin. Oncol..

[17] Grothey A., Sobrero A.F., Shields A.F., Yoshino T., Paul J., Taieb J., Souglakos J., Shi Q., Kerr R., Labianca R. (2018). Duration of Adjuvant Chemotherapy for Stage III Colon Cancer. N. Engl. J. Med..

[18] Brierley J.D., Gospodarowicz M.K., Wittekind C. (2017). TNM Classification of Malignant Tumours.

[19] Chen K., Collins G., Wang H., Toh J.W.T. (2021). Pathological Features and Prognostication in Colorectal Cancer. Curr. Oncol..

[20] Feng H., Lyu Z., Zheng J., Zheng C., Wu D.Q., Liang W., Li Y. (2021). Association of tumor size with prognosis in colon cancer: A Surveillance, Epidemiology, and End Results (SEER) database analysis. Surgery.

[21] Dai W., Li Y., Meng X., Cai S., Li Q., Cai G. (2017). Does tumor size have its prognostic role in colorectal cancer? Re-evaluating its value in colorectal adenocarcinoma with different macroscopic growth pattern. Int. J. Surg..

[22] Zhou J., Wang C., Lv T., Fan Z. (2024). Association between tumor size and prognosis in patients with small bowel adenocarcinoma-a SEER-based study. Heliyon.

[23] Guastadisegni C., Colafranceschi M., Ottini L., Dogliotti E. (2010). Microsatellite instability as a marker of prognosis and response to therapy: A meta-analysis of colorectal cancer survival data. Eur. J. Cancer.

[24] Smith H.G., Chiranth D., Mortensen C.E., Schlesinger N.H. (2022). The significance of subdivisions of microscopically positive (R1) margins in colorectal cancer: A retrospective study of a national cancer registry. Color. Dis..

[25] Moertel C.G., Fleming T.R., Macdonald J.S., Haller D.G., Laurie J.A., Goodman P.J., Ungerleider J.S., Emerson W.A., Tormey D.C., Glick J.H. (1990). Levamisole and fluorouracil for adjuvant therapy of resected colon carcinoma. N. Engl. J. Med..

[26] Mayer R.J. (2012). Oxaliplatin as part of adjuvant therapy for colon cancer: More complicated than once thought. J. Clin. Oncol..

[27] Kuebler J.P., Wieand H.S., O’Connell M.J., Smith R.E., Colangelo L.H., Yothers G., Petrelli N.J., Findlay M.P., Seay T.E., Atkins J.N. (2007). Oxaliplatin combined with weekly bolus fluorouracil and leucovorin as surgical adjuvant chemotherapy for stage II and III colon cancer: Results from NSABP C-07. J. Clin. Oncol..

